# Fracture–dislocations of the forearm joint: a systematic review of the literature and a comprehensive locker-based classification system

**DOI:** 10.1186/s10195-020-00562-8

**Published:** 2020-12-02

**Authors:** Stefano Artiaco, Federico Fusini, Arman Sard, Elisa Dutto, Alessandro Massè, Bruno Battiston

**Affiliations:** 1 Department of Orthopaedic and Traumatology, Hand and Microsurgery Unit, Orthopaedic and Trauma Centre, AOU Città Della Salute E Della Scienza Di Torino, via Zuretti 29, 10126 Turin, Italy; 2 Department of Orthopaedic and Traumatology, Orthopaedic and Trauma Centre, AOU Città Della Salute E Della Scienza Di Torino, University of Turin, via Zuretti 29, 10126 Turin, Italy

**Keywords:** Forearm joint, Forearm fracture–dislocation classification, Monteggia, Galeazzi, Essex-Lopresti

## Abstract

**Background:**

Monteggia, Galeazzi, and Essex-Lopresti injuries are the most common types of fracture–dislocation of the forearm. Uncommon variants and rare traumatic patterns of forearm fracture–dislocations have sometimes been reported in literature. In this study we systematically review the literature to identify and classify all cases of forearm joint injury pattern according to the forearm joint and three-locker concepts.

**Methods:**

A comprehensive search of the PubMed database was performed based on major pathological conditions involving fracture–dislocation of the forearm. Essex-Lopresti injury, Monteggia and Galeazzi fracture–dislocations, and proximal and/or distal radioulnar joint dislocations were sought. After article retrieval, the types of forearm lesion were classified using the following numerical algorithm: proximal forearm joint 1 [including proximal radioulnar joint (PRUJ) dislocation with or without radial head fractures], middle radioulnar joint 2, if concomitant radial fracture R, if concomitant interosseous membrane rupture I, if concomitant ulnar fracture U, and distal radioulnar joint 3 [including distal radioulnar joint (DRUJ) dislocation with or without distal radial fractures].

**Results:**

Eighty hundred eighty-four articles were identified through PubMed, and after bibliographic research, duplication removal, and study screening, 462 articles were selected. According to exclusion criteria, 44 full-text articles describing atypical forearm fracture–dislocation were included. Three historical reviews were added separately to the process. We detected rare patterns of two-locker injuries, sometimes referred to using improper terms of variant or equivalent types of Monteggia and Galeazzi injuries. Furthermore, we identified a group of three-locker injuries, other than Essex-Lopresti, associated with ulnar and/or radial shaft fracture causing longitudinal instability. In addition to fracture–dislocations commonly referred to using historical eponyms (Monteggia, Galeazzi, and Essex-Lopresti), our classification system, to the best of the authors’ knowledge, allowed us to include all types of dislocation and fracture–dislocation of the forearm joint reported in literature. According to this classification, and similarly to that of the elbow, we could distinguish between simple dislocations and complex dislocations (fracture–dislocations) of the forearm joint.

**Conclusions:**

All injury patterns may be previously identified using an alphanumeric code. This might avoid confusion in forearm fracture–dislocations nomenclature and help surgeons with detection of lesions, guiding surgical treatment.

**Level of evidence:**

V.

## Introduction

Over the last two decades, anatomical and biomechanical knowledge of the forearm has greatly improved, and some traumatic injuries involving this anatomical segment can now be seen from a new perspective.

The concept of forearm joints as described by Dumontier and Soubeyrand is a cornerstone of the full understanding of forearm injuries [1, 2]. The forearm acts as a single functional unit constituted of:

- Two bones: radius and ulna
- The interosseous membrane (IOM)
- One functional joint: the middle radioulnar joint (MRUJ), formed by the forearm bones and IOM
- Two anatomical joints: the proximal radioulnar joint (PRUJ) and distal radioulnar joint (DRUJ)

The forearm joint thus has two anatomical lockers (PRUJ and DRUJ) and one functional locker (MRUJ), allowing stability during pronation and supination of the forearm. The IOM plays a major role in forearm stability and allows load transfer from the radius to ulna. The structure of the IOM includes five ligaments: central band, accessory band, distal oblique bundle, proximal oblique cord, and dorsal oblique accessory cord [3]. The central band is the widest and thickest part of the IOM, representing the most important anatomic component of the membrane. The central tendinous portion of the IOM is obliquely oriented, forming an average angle of 20° with the longitudinal axis of the radius and 28° with the longitudinal axis of the ulna [4]. Additional ligaments, also known as accessory bands, are oriented in the same direction as the central band to complete the middle ligamentous complex of the IOM [3]. The remaining structures of the IOM (distal oblique bundle, proximal oblique cord, and dorsal oblique accessory cord) are not anatomically constant. Nonetheless, the distal IOM is considered to be a secondary stabilizer of the distal radioulnar joint when other soft tissue structures of the DRUJ are compromised [5].

The lockers may be locked, absent, or unstable, with different combinations recognized in many forearm fracture–dislocation patterns [6, 7]. Standard radiographs usually enable the identification of fracture–dislocations of the forearm joint, and/or dislocations of the radial head and caput ulnae. In contrast, diagnosis of IOM injuries in the context of forearm trauma remains challenging. Both ultrasound (US) and magnetic resonance imaging (MRI) demonstrate similar ability to recognize complete destruction of the central part of the IOM in cadaveric study [8]. As reported by Rodriguez-Martin, each technique has well-known advantages and limitations and should therefore be adapted case by case to the specific clinical situation [9].

Historically, fracture–dislocations of the forearm included Monteggia, Galeazzi, and Essex-Lopresti injuries [10–12]. These injuries were described during the nineteenth and twentieth centuries and are usually reported in literature with the corresponding eponyms. However, in a recent systematic review of literature focused on the use of eponyms in shoulder and elbow surgery, Somford et al. demonstrated that this use of eponymous terms is inadequate and inconsistent, because they are not used properly and their meaning varies from surgeon to surgeon [13].

In particular, for forearm injuries, Somford observed that:

- For Monteggia fractures, 11 (52%) articles did not clearly identify the injury. A total of five (24%) descriptions were divergent, and the remaining five (24%) had a description similar to the original one.
- For Galeazzi fractures, four (40%) articles did not clearly define a Galeazzi fracture. One (10%) description was divergent, and the remaining five (50%) had a description similar to the original one.
- For Essex-Lopresti injuries, one (9%) article did not clearly define an Essex-Lopresti injury. One (9%) description was divergent, and the remaining nine (82%) had a description similar to the original one.

Moreover, some patterns of forearm injuries similar to that of Monteggia, Galeazzi, and Essex-Lopresti have sometimes been reported in literature as “variant, like, equivalent” with inappropriate terminology that leads to subsequent confusion.

Finally, another pattern of forearm injury involving PRUJ and DRUJ dislocation with integrity of IOM was recently reported in literature [14–19]. This injury was called “crisscross” by Leung, according to the relative position of the radius and ulna visible on plain radiographs of the forearm [14].

Fracture–dislocations of the forearm have rarely been considered as a single group of injuries in literature. Only one, German-language study, published by Lendemans et al., included Monteggia, Galeazzi, and Essex-Lopresti injuries together, reporting that the feature common to all three forms is the combination of a forearm fracture with instability of the distal or proximal radioulnar joint [20].

In the light of the recent concept of the forearm joint and the three-locker system, we systematically review herein the literature on Monteggia, Galeazzi, and Essex-Lopresti injuries and their variants and equivalent injuries. We further review the literature for isolated and/or combined proximal and distal radioulnar dislocation with or without associated forearm fractures to identify any cases of forearm joint injury patterns that are not included in historical descriptions.

A classification system based on the involvement of forearm lockers might overcome the inaccuracies related to the eponymous nomenclature.

The aim of this study is to detect the possible atypical patterns of fracture–dislocations of the forearm reported in literature and provide a comprehensive classification system for them for potential use as a tool to guide surgical treatment.

## Materials and methods

To provide a new classification of forearm joint fracture–dislocations, a comprehensive search of the PubMed database was carried out based on the major pathological conditions involving fracture–dislocation of the forearm. Essex-Lopresti injury, Monteggia and Galeazzi fracture–dislocations, and proximal and/or distal radioulnar joint dislocations were sought. Since Essex-Lopresti, Monteggia, and Galeazzi fracture–dislocations are already well described, the search mainly focused on the retrieval of other, atypical patterns of fracture–dislocation. The database search was conducted from 1998 until August 2018. All clinical studies, clinical trials, and case reports were included. Articles written in languages other than Italian, English, French, and German were excluded. All article titles and abstracts were firstly reviewed by two authors (S.A. and F.F.) independently. The reference lists of all included articles were screened to identify any additional original articles. The search was limited to adult human subjects. Only three historical reviews regarding Essex-Lopresti, Monteggia, and Galeazzi fracture–dislocations were included, to describe them in our classification system [10–12].

After identification, articles were analyzed for the description of forearm fracture–dislocation patterns, and each pattern was classified according to the anatomical structures involved. No analysis was carried out to ascertain the quality of research or interpret the results of the articles.

**Fig. 1:**
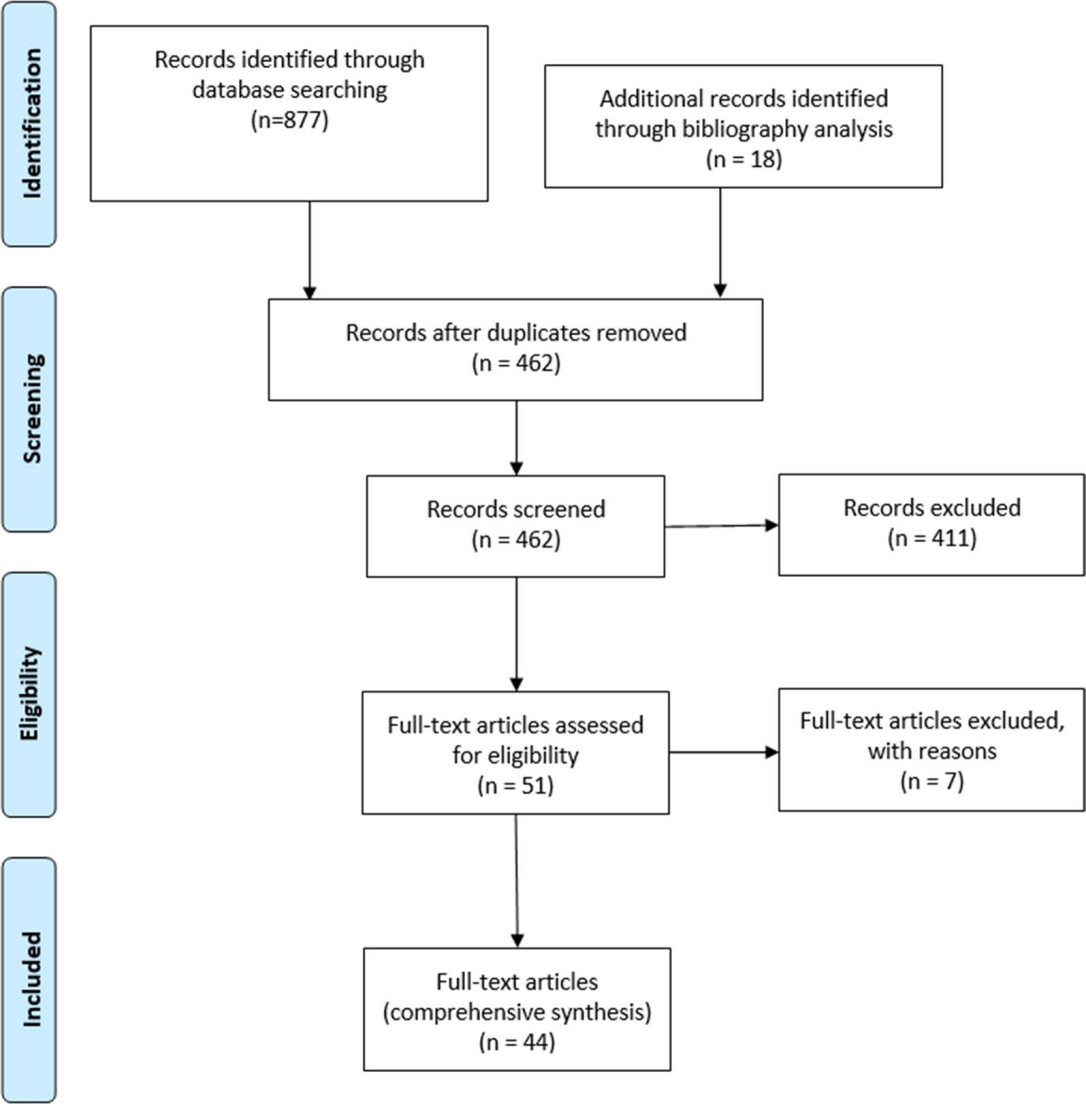
Preferred Reporting Items for Systematic Review and Metaanalysis (PRISMA) flow diagram of articles retrieved, screened, and selected through the database search

A flow diagram describing the search is shown in Fig. 1.

After article retrieval, the types of forearm lesion were classified using the following numerical algorithm:

– Proximal forearm joint 1 (including PRUJ dislocation with or without radial head fracture)

– Middle radioulnar joint 2, if concomitant radial fracture R, if concomitant interosseous membrane rupture I, if concomitant ulnar fracture U

– Distal radioulnar joint 3 (including DRUJ dislocation with or without distal radial fractures)

## Results

A total of 884 articles were identified in PubMed and by bibliographic research (135 Galeazzi, 322 Monteggia, 150 Essex-Lopresti, 187 radial head dislocation, 72 ulnar head dislocation, 1 PRUJ dislocation, and 17 DRUJ dislocation). After duplication removal and study screening, 462 articles were selected. According to the exclusion criteria, 44 full-text articles describing atypical forearm fracture–dislocation were included (9 Galeazzi, 8 Monteggia, 5 Essex-Lopresti, 7 radial head dislocation, 0 ulnar head dislocation, 1 PRUJ dislocation, and 4 DRUJ dislocation). Three historical reviews were added separately [10–12].

### Classification system

Single-locker injuries do not cause dislocations of the forearm and were not included in this systematic review. Dislocations or fracture–dislocations, occurring when two or three lockers of the forearm joint are involved, were included in the comprehensive classification system.

According to our classification, 13 combinations of forearm fracture–dislocations of the forearm joint are possible (Table 1).

**Table 1:** Description of possible combinations of forearm fracture–dislocation patterns. Each lesion is described based on the anatomical structures involved in each type of forearm fracture–dislocation

Types	1 (PRUJ)	2 (MRUJ)			3 (DRUJ)	Notes
	PRUJ dislocation	IOM rupture	Ulnar fracture	Radial fracture	DRUJ dislocation	
Two-locker injuries						
1.2I	×	×				Isolated radial head fracture
1.2IU	×	×	×			Monteggia fracture dislocation
1.2IR	×	×		×		
1.2IRU	×	×	×	×		
2I.3		×			×	Isolated dislocation of ulnar head
2IR.3		×		×	×	Galeazzi injury
2IU.3		×	×		×	Never described in literature
2IRU.3		×	×	×	×	
1.3	×				×	Leung crisscross injury
Three-locker injuries						
1.2I.3	×	×			×	Essex-Lopresti injury
1.2IRU.3	×	×	×	×	×	
1.2IR.3	×	×		×	×	
1.2IU.3	×	×	×		×	

1 proximal locker, 2 middle locker, 3 distal locker, I interosseous membrane, U ulnar fracture, R radial fracture, PRUJ proximal radioulnar joint, IOM interosseous membrane, DRUJ distal radioulnar joint

The systematic review of the literature identified reports on all but one possible combination. The only pattern of fracture–dislocation of the forearm with two- or three-locker injury that was not identified in this systematic review of the literature was 2IU.3.

## Discussion

**Fig. 2:**
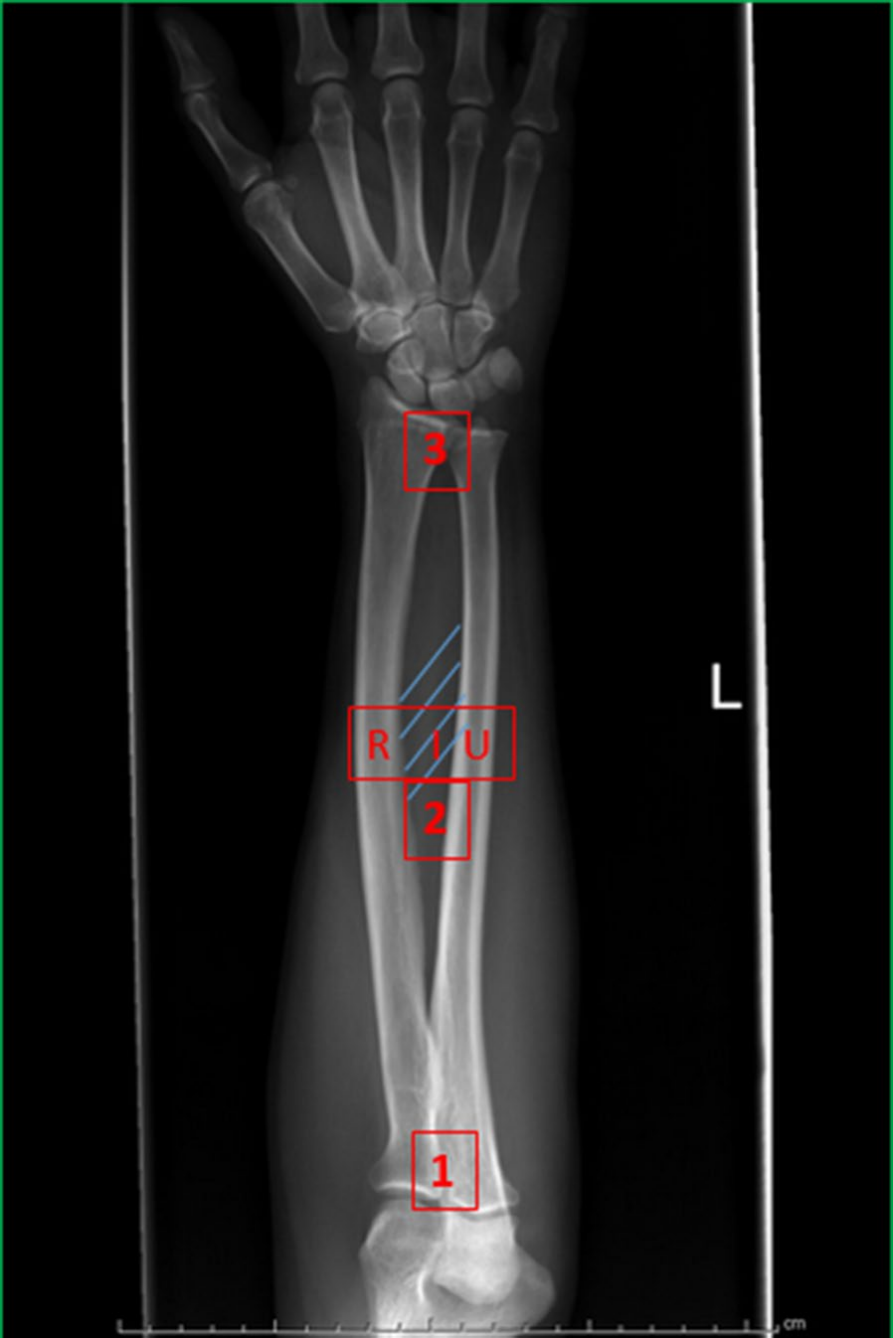
Visualization of the three lockers involved in the stability of the forearm: proximal radioulnar joint (PRUJ), middle radioulnar joint (MRUJ) composed of interosseous membrane (IOM), radius shaft (R), and ulnar shaft (U), distal radioulnar joint (DRUJ)

As reported by Dumontier and Soubeyrand, the forearm joint has three main functions: to allow pronosupination and positioning of the hand, to transfer and share loading stress among the forearm bones, and to serve as an attachment site for forearm muscle [1, 2]. PRUJ and DRUJ are anatomical lockers, while MRUJ, constituted by the radius and ulna shaft together with the IOM, is a functional locker intercalated between the former two (Fig. 2) [21].

Forearm rotation follows the axis directed from the radial head to the ulnar fovea, occurring with full range of motion and physiological stability when forearm bones and soft tissues stabilizers of PRUJ, MRUJ, and DRUJ are preserved [22]. Recent findings have suggested that PRUJ and MRUJ are probably more critical than IOM for forearm rotation, while IOM provides static longitudinal stabilization of the forearm and less so as a rotational stabilizer [23].

Absence of a single locker has little if any consequence on longitudinal stability, but when two lockers are unstable, the third cannot compensate. Furthermore, when three lockers are unstable, longitudinal instability occurs, such as in Essex-Lopresti injury.

Many fracture–dislocations of the forearm joint, including Monteggia, Galeazzi, Essex-Lopresti, isolated dislocation of the ulnar head, and Leung crisscross injury, have previously been listed in the same group of injuries because they are linked by the concept that two or three lockers are damaged [1, 24].

However, our systematic review of the literature shows that other patterns of fracture–dislocations of the forearm may rarely occur and that all these injuries, together with those previously reported, may be included in a single classification scheme on the basis of the forearm joint concept and three-locker system described by Dumontier and Soubeyrand [1, 2].

According to our classification system, two- and three-locker injuries can be distinguished (Table 2).

**Table 2:** Pattern description of forearm fracture–dislocations and list of authors reporting each type of injury

Type	Lesion description	Authors
Two-locker injuries		
1.2I	PRUJ dislocation–IOM rupture	Rethnam [23], Sharma [24], Obert [25], El Ibrahimi [28], Watanabe [27], Koulali-Idrissi [29]
1.2 IU	PRUJ dislocation–IOM rupture–ulna fracture	Rehim [5]
1.2IR	PRUJ dislocation–IOM rupture–radius fracture	Rao [38], Haddad [39], Linzel [40], Mehara [41], Cherif [42], Simpson [45], Shamian [43], Singh [44]
1.2RIU	PRUJ dislocation–IOM rupture–ulna fracture–radius fracture	Ouakrim [63]
2I.3	IOM rupture–DRUJ dislocation	Wassink [30], Szabo [31], Bruckner [32], Carlsen [33]
2IR.3	IOM rupture–radius fracture–DRUJ dislocation	Sebastin [6]
2 IU.3	IOM rupture–ulna fracture–DRUJ dislocation	None
2RIU.3	IOM rupture–radius fracture–ulna fracture–DRUJ dislocation	Ryan [64], Vaishya [62]
1.3	PRUJ dislocation–DRUJ dislocation	Leung [9], Verettas [10], Potter [11], Nishi [12], Spicer [13], Papageorgiu [14], Wong [35], Tosun [36], Raghavendra [37]
Three-locker injuries		
1.2I.3	PRUJ dislocation–IOM rupture–DRUJ dislocation	McGlinn [7]
1.2RIU.3	PRUJ dislocation–IOM rupture–radius fracture–ulna fracture–DRUJ dislocation	Koutserimpas [57], Mann [55], Kanso [56], Rappold [58], Clare [59], Letta [60]
1.2IR.3	PRUJ dislocation–IOM rupture–radius fracture–DRUJ dislocation	Kedous [48], Jones [49], Khurana [50], Eglseder [51], Falsafi [52]
1.2IU.3	PRUJ dislocation–IOM rupture–ulna fracture–DRUJ dislocation	Cheung [53], Jafari [54]

*PRUJ* proximal radioulnar joint, *IOM* interosseous membrane, *DRUJ* distal radioulnar joint

### Two-locker injuries

Fracture–dislocations of the forearm joint involving two lockers include nine possible patterns.

Monteggia (1.2 IU) and Galeazzi (2IR.3) injuries are the most common pattern of fracture–dislocations of the forearm belonging to this group. According to the historical description, the Monteggia injury is a fracture of the shaft of the ulna associated with a dislocation of the radial head [10], while the Galeazzi injury is a fracture of the radius shaft associated with dislocation of the radial head [11].

We believe that the eponyms Monteggia and Galeazzi should be used exclusively to identify forearm fracture–dislocations corresponding to the original description.

However, over time, many forearm fracture–dislocations with a similar pattern have been reported in literature and variably named Monteggia or Galeazzi as equivalent or variant injuries. Sometimes this nomenclature is not properly used, thus generating misunderstanding and confusion.

From this analysis of literature, disagreement is evident over Monteggia-like injuries. In 1967, Bado introduced the concept of Monteggia lesions and classified four types of injury according to their radiological appearance [25]. The Bado type II, represented by a Monteggia lesion with posterior dislocation of the radial head and ulna fracture, may include some patterns of so-called Monteggia-like injuries with complex fracture–dislocation of the ulnohumeral joint. These injuries are very different from the anatomopathological findings and surgical treatments of the original pattern described by Monteggia and should not be confused.

With regard to Monteggia-like injuries, we believe that the term should be reserved for forearm joint injuries similar to the original pattern. We agree with Laun and Jungbluth, who defined this injury as a fracture of the proximal ulna distal to the olecranon with associated dislocation and fracture of the radial head, alone or combined with a fracture of the coronoid process [26, 27]. In our opinion, the use of the eponymous terms Monteggia or Galeazzi for equivalent or variant injuries has not yet been sufficiently defined and thus should be avoided or used with care.

Isolated dislocation of the radial head (1.2I) or ulnar head (2I.3) without forearm fractures are the patterns of the other two-locker injuries. They may occur when the proximal and middle part of the IOM is damaged together with PRUJ, or when the distal and middle part of the IOM is involved together with DRUJ.

In a cadaveric study, Hayami demonstrated that isolated radial head dislocation (defined as more than 50% displacement) was possible when annular and quadrate ligaments and the proximal half of the IOM are sectioned [28]. In clinical practice, isolated radial head dislocation has frequently been reported in children and skeletally immature patients, but it is very rare in adults, with about 30 cases reported in literature [29–35].

Isolated dislocation of the ulnar head is more common than proximal dislocation of the radial head [36,37, 38,39]. This injury may occur when the triangular fibrous cartilage complex (TFCC) and distal part of the IOM are damaged because, as described by Moritomo, the distal oblique band (DOB) is a secondary stabilizer of the DRUJ [40].

In a cadaveric study, Watanabe investigated the IOM contribution to DRUJ constraint, evaluating DRUJ stability with a progressive section of DRUJ structures, distal IOM, central IOM, and proximal IOM, demonstrating that, with the distal and central IOM section, the radial head was dislocated in supination, neutral position, and pronation [33].

Simultaneous dislocation of the radial and ulnar head with intact IOM (1.3) is very rare and commonly referred to as crisscross injury. Leung reported four cases of PRUJ and DRUJ dislocation associated with radial head fracture, explaining the traumatic mechanism of ulnar and radial displacement around a pivot point represented by the intact MRUJ [14]. Seven cases of similar injuries with simultaneous dislocation of PRUJ and DRUJ without radial head fracture are reported in literature, representing another type of the same injury pattern [15–19,41,42]. Finally, a third type with PRUJ and DRUJ dislocation associated with distal radius fracture is reported only once in literature [43].

Finally, fracture–dislocations of the forearm joint with the involvement of two lockers include some rare patterns occasionally described in single case reports or small clinical series in literature.

To the best of the authors’ knowledge, ulnar and radial shaft fractures with PRUJ dislocation (1.2RIU) were reported by Oukrin, being referred to as Monteggia variants.

Ulnar and radial shaft fractures with DRUJ dislocation (2RIU.3) were reported by Vaishya (six cases) and Ryan (one case), and referred to as Galeazzi-like fractures.

Radial head dislocation and radial shaft fracture (1.2IR) was first described by Rao and Simpson in 1991 in two different publications. Subsequently, this injury pattern was reported by Linzel in a small clinical series and by Mehara, Haddad, Cherif, Simpson, Shamian, and Singh as single clinical cases, for a total of 12 cases reported in literature [44–51].

To the best of the authors’ knowledge, the last possible combination of two-locker injury, represented by ulnar shaft fractures and ulnar head dislocation (2 IU.3), has never been reported in literature. We are not able to ascertain whether this pattern could occur without concomitant injuries, but ulnar shaft fractures and ulnar head dislocation have been observed in other two- and three-locker combination patterns investigated in our systematic review (2RIU.3, 1.2RIU.3, and 1.2IU.3).

### Three-locker injuries

Essex-Lopresti injury is a pattern of forearm joint lesion characterized by fracture of the radial head with combined PRUJ, IOM (MRUJ), and DRUJ disruption (1.2I.3). These injuries are rare, accounting for about 1% of all radial head fractures. They are sometimes difficult to diagnose in acute phases and difficult to treat because all three lockers are involved, causing longitudinal instability of the forearm. The pattern of injury was clearly defined by Essex-Lopresti in 1951, and subsequently this injury has commonly been reported in literature with the eponymous term. Somford reported that most papers reported in literature agree in the description of anatomopathological findings, thus the eponymous term exactly identifies this pattern of forearm joint lesion [13]. Some variants of Essex-Lopresti injury have been rarely reported in literature. Hii described an Essex-Lopresti injury with distal displacement of the radius, and Auyeung reported another case with bony distal radioulnar joint injury [52, 53]. Both lesions were slightly different from Essex-Lopresti’s definition, but they can be included in the same pattern in the locker-based classification system.

Finally, fracture–dislocations of the forearm joint with the involvement of three lockers include some rare patterns occasionally described as single case reports or small clinical series in literature.

These injuries are characterized by ulnar and/or radial fractures and PRUJ and DRUJ dislocation causing radioulnar dissociation and longitudinal instability, as recorded in Essex-Lopresti injury.

To the best of the authors’ knowledge, PRUJ dislocation with IOM rupture, radius fracture, and DRUJ dislocation (1.2IR.3)—a very rare pattern of forearm joint fracture–dislocation—has only been reported in five cases in literature, by Kedous, Jones, Khurana, Falsafi, and Eglseder [54–58].

In the same way, PRUJ dislocation with IOM rupture, ulna fracture, and DRUJ dislocation (1.2 IU.3) is very rare, and only two patients with this kind of injury have been reported in literature, by Cheung and Jafari [59, 60].

The last combination of three-locker injury is represented by ulnar and radial shaft fractures with concomitant PRUJ and DRUJ dislocation (1.2RIU.3). Six cases of this injury are reported in literature, being named as combinations of Monteggia and Galeazzi injuries in the same forearm, by Mann, Kanso, Koutserimpas, Rappold, Letta, and Clare [61–66].

### Treatment options

Treatment options should be based on the concept that, when two lockers are unstable, the third cannot compensate, and when three lockers are unstable, longitudinal instability occurs [1].

Therefore, regarding surgical treatment, the following considerations may be suggested:

At least one locker should be reconstructed when two lockers are involved, because two out of three lockers are needed for forearm joint function.

**Fig. 3:**
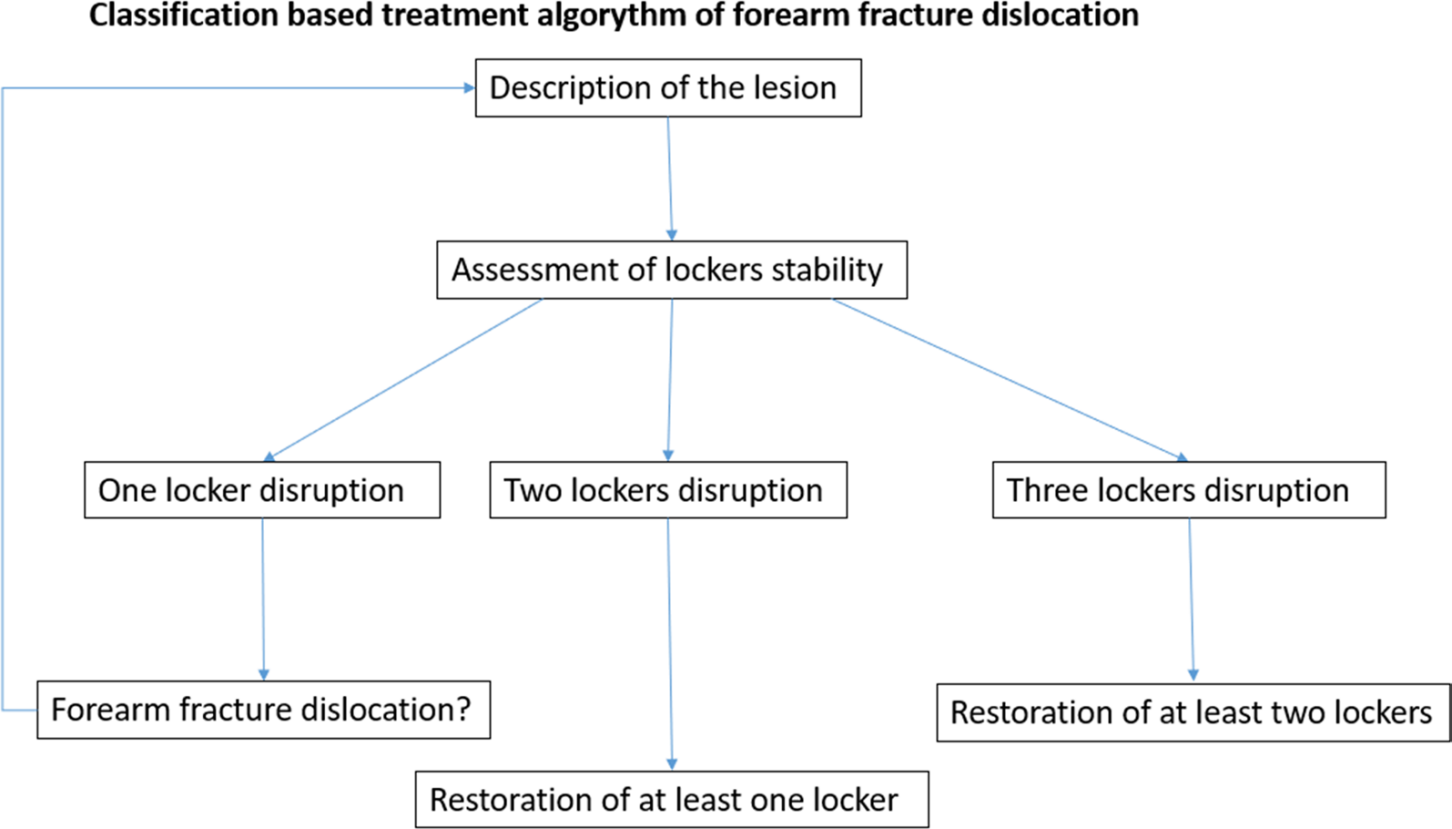
Diagnostic therapeutic flowchart for classification and treatment of forearm fracture–dislocation based on the three lockers described by Dumontier

At least two lockers should be reconstructed when three lockers are involved, to avoid longitudinal instability (Fig. 3).

In two-locker injury without fracture of forearm bones (1.2I, 2I.3, and 1.3), there is still no consensus regarding management, although after treatment, two out of three lockers should be stable to preserve adequate forearm joint function. Werthel, on the basis of a review of the literature, proposed nonoperative management for isolated dislocations of the ulnar head [67], but in nonreducible dislocation with soft-tissue interposition, open reduction, and TFCC, repair has been advocated [39]. In proximal radial head dislocations (1.2I), Obert reported closed reduction of radial head without recurrence in most adult patients. Open reduction and ligamentous repair was performed only in the case of unsuccessful conservative treatment. Regarding crisscross injury with simultaneous PRUJ and DRUJ fracture–dislocations (1.3), treatment options have been variable, including closed reduction and immobilization [19, 41], closed reduction and percutaneous DRUJ pinning [15, 18], and open reduction with radial head fixation or ligamentous repair [14, 16, 17].

In two-locker injuries with ulna and/or radius shaft fracture, forearm bones must undergo osteosynthesis. In these cases, radial or ulnar shaft fixation is sufficient to repair MRUJ, while the IOM does not require reconstruction, as in Monteggia (1.2 IU) and Galeazzi (2IR.3) injuries.

After fixation of Monteggia and Galeazzi fractures, PRUJ and DRUJ should be evaluated to confirm reduction and stability of radial and ulnar head. In the case of residual PRUJ and DRUJ dislocation or instability, temporary fixation (PRUJ) or open reduction and ligamentous repair (PRUJ or DRUJ) should be performed.

Cases of the remaining two-locker injuries with ulna and/or radius shaft fractures (1.2RIU, 2RIU.3, and 1.2IR) are very rare, and literature data are insufficient to define standard treatment [44–51, 68–70]. Nonetheless, we believe that fracture fixation should be performed first, and subsequent PRUJ and DRUJ stability should be evaluated to determine further treatment.

In three-locker injuries, at least two lockers must be reconstructed.

In Essex-Lopresti injury (1.2I.3), PRUJ and DRUJ should always be reconstructed. Radial head fixation or radial head prosthetic replacement should be established according to the characteristic of the radial head fracture.

DRUJ reconstruction or fixation may allow stabilization of the second locker, thus avoiding longitudinal instability. At present, although there is not yet a shared management scheme for these injuries, repair of the third locker by means of IOM reconstruction is commonly performed in association with proximal and distal locker reconstruction [7, 71–76]. IOM reconstruction may in fact allow adequate load transmission along the forearm bones, improving the overall stabilization of the forearm joint.

In three-locker injuries with ulna and/or radius shaft fractures (1.2RIU.3, 1.2RI.3, and 1.2IU.3), the forearm bones must first undergo osteosynthesis, stabilizing MRUJ. After fracture fixation, PRUJ and DRUJ should be evaluated to confirm reduction and stability of radial and ulnar head, and eventually repaired in the case of residual instability. In these rare injuries, fracture fixation and PRUJ and/or DRUJ repair is the usual treatment. IOM reconstruction has never been reported in these circumstances [54–57, 59–61, 63, 66, 77].

**Fig. 4:**
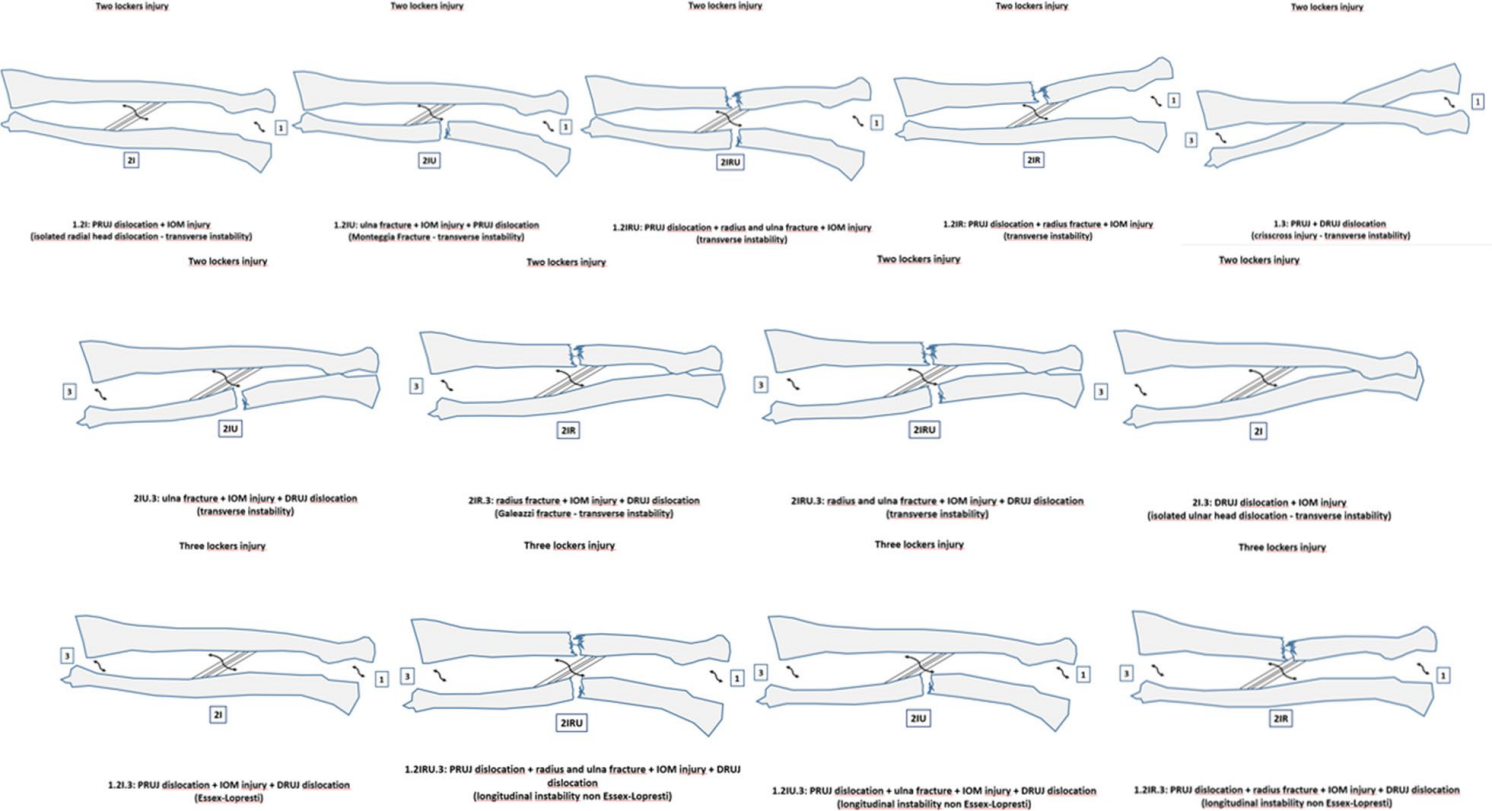
Illustration of different patterns of fracture described in Table 2

All the types of fracture–dislocations are illustrated in Fig. 4.

## Conclusions

Fracture–dislocations of the forearm joint are commonly reported in literature using eponymous terms. Nonetheless, it is clear that a classification based on eponyms is not always accurate and is insufficient to include all patterns of fracture–dislocations of the forearm.

Through a systematic review of the literature, we detected rare patterns of two-locker injuries sometimes referred to by incorrect terms of variant or equivalent types of Monteggia and Galeazzi injuries.

Furthermore, we identified a group of three-locker injuries, other than Essex-Lopresti, associated with ulnar and/or radial shaft fractures, causing longitudinal instability of the forearm.

In addition to fracture–dislocations commonly referred to by historical eponyms (Monteggia, Galeazzi, and Essex-Lopresti), to the best of the authors’ knowledge, our classification system allowed us to include all types of dislocations and fracture–dislocations of the forearm joint reported in literature.

According to this classification, and similarly to that of the elbow, we could distinguish between simple dislocations and complex dislocations (fracture–dislocations) of the forearm joint.

All the injury patterns may be previously identified using an alphanumeric code. This might avoid confusion in forearm fracture–dislocation nomenclature and help surgeons in lesion detection, thus guiding surgical treatment.

## Data Availability

All relevant data for the research are included in the manuscript as Table 2
